# The impact of genetic risk for schizophrenia on eating disorder clinical presentations

**DOI:** 10.1038/s41398-023-02672-3

**Published:** 2023-11-29

**Authors:** Ruyue Zhang, Ralf Kuja-Halkola, Stina Borg, Virpi Leppä, Laura M. Thornton, Andreas Birgegård, Cynthia M. Bulik, Sarah E. Bergen

**Affiliations:** 1 Department of Medical Epidemiology and Biostatistics, Karolinska Institutet, Stockholm, Sweden; 2 Department of Genetics, University of North Carolina at Chapel Hill, Chapel Hill, USA; 3 Department of Psychiatry, University of North Carolina at Chapel Hill, Chapel Hill, USA; 4 Department of Nutrition, University of North Carolina at Chapel Hill, Chapel Hill, USA

**Keywords:** Clinical genetics, Human behaviour

## Abstract

A growing body of literature recognizes associations between eating disorders (EDs) and schizophrenia and suggests that familial liability to schizophrenia in individuals with anorexia nervosa (AN) reveals distinct patterns of clinical outcomes. To further investigate the influence of schizophrenia genetic liability among individuals with EDs, we evaluated the associations between schizophrenia polygenic risk scores (PRS) and clinical presentations of individuals with EDs including their overall health condition and ED-related symptoms. Using data from two previous studies of the genetics of EDs comprising 3,573 Anorexia Nervosa Genetics Initiative (ANGI) cases and 696 Binge Eating Genetics Initiative (BEGIN) cases born after 1973 and linked to the Swedish National Patient Register, we examined the association of schizophrenia PRS on ED clinical features, psychiatric comorbidities, and somatic and mental health burden. Among ANGI cases, higher schizophrenia PRS was statistically significantly associated with higher risk of major depressive disorder (MDD) measured by hazard ratio (HR) with 95% confidence interval (CI) (HR [95% CI]: 1.07 [1.02, 1.13]) and substance abuse disorder (SUD) (HR [95% CI]: 1.14 [1.03, 1.25]) after applying multiple testing correction. Additionally, higher schizophrenia PRS was associated with decreased clinical impairment assessment scores (−0.56, 95% CI: [−1.04, −0.08]) at the conventional significance level (*p* < 0.05). Further, in BEGIN cases, higher schizophrenia PRS was statistically significantly associated with earlier age at first ED symptom (−0.35 year, 95% CI: [−0.64, −0.06]), higher ED symptom scores (0.16, 95% CI: [0.04, 0.29]), higher risk of MDD (HR [95% CI]: 1.18 [1.04, 1.34]) and SUD (HR [95% CI]: 1.36 [1.07, 1.73]). Similar, but attenuated, patterns held in the subgroup of exclusively AN vs other eating disorder (OED) cases. These results suggest a similar pattern of influence of schizophrenia PRS for AN and OED cases in terms of psychiatric comorbidities, but a different pattern in terms of ED-related clinical features. The disparity of the effect of schizophrenia PRS on AN vs OED merits further investigation.

## Introduction

Eating disorders (EDs) are severe and often protracted illnesses with complex clinical manifestations and frequent psychiatric comorbidity—most notably obsessive-compulsive disorder (OCD) 22–32%, anxiety disorders 37–56%, and major depressive disorder (MDD) 36–68% [1, 2]. Multiple somatic complications are also associated with EDs, which often lead to poor functioning and premature mortality [3–6]. In fact, the mortality rate for anorexia nervosa (AN) is the second highest among all mental disorders, and the recovery rates among adults are only 30–50% [7–10]. More than 20% of individuals continue to have AN at long-term follow-up and develop an enduring course with poor outcomes [7]. In contrast, the recovery rate for bulimia nervosa (BN) is generally more favorable, 60–75% [9, 11]. However, the relapse rate is high for both AN and BN at 30–40% of treated patients [11–13]. Moreover, frequent diagnostic migration across ED presentations further complicates the clinical picture [14]. There is a pressing need for research on the underlying etiology of the varying clinical presentation clusters in service of more targeted and effective treatment planning.

Accumulating evidence supports an association between EDs and schizophrenia. For example, significant familial co-aggregation of EDs and schizophrenia has been reported in recent large-scale investigations, indicating that both illnesses share genetic and environmental causes [15]. Significant positive single-nucleotide polymorphism (SNP)-based genetic correlations of 0.19–0.29 provide additional evidence of genetic overlap between AN and schizophrenia [16, 17]. These observations prompted the hypothesis that familial factors for schizophrenia might play a role in the development of EDs. Moreover, a recent study demonstrated that family history of schizophrenia is associated with greater risk of multiple psychiatric comorbidities, greater cumulative somatic and mental health burden, and increased suicide risk, but less severe ED-related symptoms in individuals with AN [18]. Alternative analytic approaches are required to further clarify the nature and extent of contributions of schizophrenia genetic liability to various EDs.

The schizophrenia polygenic risk score (PRS) combines the weighted effect of a large number of SNPs that distinguish schizophrenia cases from healthy controls [19]. The observed variance explained by the PRS has increased from 3.2% to 22.1% as the sample sizes of the genome-wide association studies (GWAS) used to derive them have increased [19–21]. Thus, PRS can be used as a tool to estimate and index the genetic liability to schizophrenia in the investigation of genetic contributions to various traits. Substantial heterogeneity exists in both the clinical presentation and outcome of EDs. Examining the impact of schizophrenia PRS on the clinical presentation of EDs has the potential to reveal how these genetic contributions are expressed and yield significant diagnostic, prognostic, and therapeutic implications.

Here, we extended previous work by investigating the influence of schizophrenia genetic liability on clinical presentation among individuals with different EDs, their overall health condition, and ED-related symptoms using linked longitudinal phenotypic and genetic data. Specifically, we examined the effect of schizophrenia PRS on age of ED onset, ED-specific symptoms and severity, risk of multiple psychiatric comorbidities, and cumulative somatic and mental health burden in individuals with EDs.

## Methods

### Data source and study population

The Anorexia Nervosa Genetics Initiative (ANGI) and Binge Eating Genetics INitiative (BEGIN) are international collaborations with large samples of genotyped cases and controls with AN and other eating disorders (OED), respectively [22, 23]. The recruitment procedures and genetic analytic methods for ANGI and BEGIN have been described previously [22, 23]. In brief, case participants from Sweden were recruited into ANGI and BEGIN via Swedish treatment centers and the National Eating Disorder Quality Registers (Riksät and Stepwise) [22, 24]. Case definitions for ANGI established a lifetime DSM-IV-based diagnosis of AN via responses to the ED100K-v1 questionnaire [22]. Importantly, ANGI cases may also have received an additional OED diagnosis given that diagnostic crossover is common in EDs [14]. For BEGIN, individuals with DSM-IV-based diagnoses of BN, atypical BN, or binge-eating disorder from Riksät or via algorithm based on responses from the ED100K-v2 were classified as other eating disorder (OED) cases. In addition, BEGIN cases may also have received an AN diagnosis. Thus, the AN and OED groups are not mutually exclusive. In addition to survey data, the Swedish samples were linked to the Swedish National Patient Register (NPR) which provided further information on inpatient and outpatient records [25]. These diagnostic records were used to define outcomes including psychiatric comorbidities, cumulative somatic and mental health burden, as well as total hospital contacts and inpatient days related to EDs.

The Swedish arms of ANGI and BEGIN were approved by the regional Ethical Review Board in Stockholm (Dnr 2013/112-31/2, 2014/1563-32, 2015/693-32, 2015/746-32, 2015/932-32, 2015/982-32, 2016/0404-32, 2016/1852-32, 2017/1352-32, 2017/0040-32, 2018/1584-32, 2018/1943-32, 2019-02779).

Our study population includes all Swedish ANGI cases and Swedish BEGIN (Freeze 1) cases born after 1973 with available NPR linkage. After excluding individuals with mismatched genetic sex and reported sex, those with very low or high genetic heterogeneity, those with high familial relatedness (pi_hat > 0.2), and ancestry outliers, the final sample comprised 3573 ANGI cases (age 18–45 years; 77 males) and 696 BEGIN cases (age 16–45 years; 18 males) with follow-up through December 31, 2018.

Although ANGI and BEGIN recruited cases primarily based on their AN or OED diagnosis respectively, diagnostic crossover is common (i.e., ANGI cases were recruited primarily based on their AN diagnoses, but 55.1% also have OED diagnoses in their lifetime, whereas BEGIN cases were recruited primarily based on their OED diagnoses, but 43.7% also have AN diagnoses in their lifetime). In order to further explore diagnostic differences, we conducted sensitivity analyses (Supplementary Table S2) with 1606 AN cases with no history of OED and 392 OED cases with no history of AN.

### Schizophrenia PRS

PRS for schizophrenia were computed for each participant in the study population using PLINK 2.0 and the summary statistics from the latest Psychiatric Genomics Consortium (PGC) schizophrenia Genome-wide association study (GWAS) of 69,369 people with schizophrenia and 236,642 controls as the discovery dataset [21]. Detailed information on genotyping the ANGI and BEGIN samples was previously reported [22, 23].

SNP quality control, including filtering for minor allele frequency (MAF > 0.10), removing duplicate SNPs and matching with the discovery dataset [26], resulted in a subset of 190,786 SNPs for ANGI and 153,212 SNPs for BEGIN. These were then clumped into clusters of approximate linkage disequilibrium (*R*^2^ < 0.25, within 200 kb distance). The classic thresholding + clumping approach was used to generate PRS at different *p* value thresholds (*p* values: 5e−08, 1e−06, 1e−04, 0.001, 0.01, 0.05, 0.1, 0.2, 0.5, 1) [27]. Results presented in this study utilize PRS scores based on the first principal component, PRS-PC1, from the principal component analysis (PCA) on the set of ten PRS in R, version 4.0.5 [28].

### Assessment of outcomes

Outcomes of interest for this study were obtained from answers to ANGI and BEGIN surveys and the linked Swedish NPR records, including clinical features of EDs, psychiatric comorbidities, and cumulative somatic and mental health burden.

#### Clinical features of EDs

For ANGI participants, clinical features of EDs include age at first ED-related symptom, the lowest illness-associated body mass index (BMI), total number of hospital contacts related to EDs, total inpatient days related to EDs, and ED severity as measured by the Clinical Impairment Assessment (CIA) scores at the recruitment of ANGI. CIA is a 16-item questionnaire which measures the severity of secondary psychosocial impairment due to ED features with higher scores indicating greater impairment. Age at first ED symptom was obtained from either the youngest age of any self-reported ED symptom including lowest BMI after reaching adult height from the ANGI survey or NPR records of AN, as available. When both were available, the earlier age was selected. The lowest illness-associated BMI was obtained from the ANGI survey. The total number of hospital contacts related to EDs included all in- and outpatient records (but not primary care records) related to EDs (relevant ICD diagnostic codes are presented in sTable 1) with both primary and secondary diagnoses available in the NPR. Total inpatient days related to EDs were calculated from the sum of all inpatient days with any ED diagnosis as a primary or secondary diagnosis.

For BEGIN participants, clinical features of EDs included age at first ED-related symptom, the lowest BMI after reaching adult height, total number of hospital contacts related to EDs, total inpatient days related to EDs, and current ED severity as measured by the Eating Disorder Examination Questionnaire (EDE-Q 6.0) [29]. The EDE-Q includes restraint, eating concern, weight concern, and shape concern subscales at BEGIN recruitment, and was scored according to published recommendations [30]. Age at first ED-related symptom was obtained from the BEGIN survey and was defined as the earliest age (after age 6 years) of ED-related symptoms including binge eating; vomiting; fasting; using laxatives, diuretics, or diet pills; and age at lowest illness-associated BMI. The total number of hospital contacts related to EDs and total inpatient days related to EDs were defined the same way as for the ANGI participants as described previously. EDE-Q subscale scores (restraint, eating concern, weight concern, shape concern) and global score were obtained from BEGIN participants at enrolment; higher scores indicate more ED pathology.

#### Psychiatric comorbidities

Psychiatric comorbidities (including attention-deficit/hyperactivity disorder (ADHD), substance abuse disorder (SUD), autism spectrum disorder (ASD), MDD, OCD, schizophrenia, and anxiety disorders) and the date of initial diagnosis for each disorder were identified from the NPR and the cause of death register for each participant using the ICD diagnostic codes presented in sTable 1.

#### Cumulative somatic and mental health burden

The total number of separately entered primary and secondary diagnoses (i.e., each diagnostic code may be counted multiple times), total number of all unique diagnoses (i.e., each diagnostic code was counted only once), and total inpatient duration from the NPR were used to index cumulative somatic and mental health burden. All inpatient and outpatient ICD records with primary and secondary diagnoses from NPR received from age 6 until the end of follow-up were counted. The total inpatient duration was defined as the number of days spent in the hospital for any reason during the follow-up period.

All cumulative outcomes including total diagnoses, total unique diagnoses, total inpatient durations, total hospital contacts related to EDs (i.e., all hospital contacts with primary and secondary ED diagnoses), and total inpatient days due to EDs (i.e., inpatient days with EDs as primary discharge diagnosis) were counted within specific age periods; age <12, 12–18, 19–25, and ≥26.

### Statistical analyses

Data management was performed using SAS, version 9.4 (SAS Institute, Inc.). PRS were generated using PLINK2, and all analyses were performed using R, version 4.0.5 [31].

Associations of the schizophrenia PRS-PC1 with age at first ED-related symptom, lowest illness-associated BMI, and ED severity as measured by CIA scores or EDE-Q subscale and global scores in ANGI and BEGIN cases were tested via linear regressions adjusting for birth year, sex, and the first 10 ancestry-informative principal components (PCs). Regression coefficients with corresponding *p* values and 95% confidence intervals (CIs) were reported as risk estimates per every standard deviation increase of schizophrenia PRS.

Cox regression models were used for testing the association between schizophrenia PRS and psychiatric comorbidities including ADHD, ASD, OCD, MDD, schizophrenia, SUD, and anxiety disorders in the study population with age as the underlying timescale. Individuals were followed from age 6 years until death or December 31, 2018. Birth year, sex, and the first 10 ancestry-informative PCs were adjusted in the model. Hazard ratios (HRs) with 95% CIs were reported as risk estimates for psychiatric comorbidities per standard deviation increase in schizophrenia PRS.

Time-split Poisson regressions were used for cumulative somatic and mental health burden and total hospital contacts related to EDs and total inpatient days related to EDs with adjustment for the first 10 ancestry-informative PCs, birth year, sex, and different time periods (age <12, 12–18, 19–25, and ≥26 years). Regression coefficients or incidence rate ratios (IRRs) with corresponding *p* values and 95% CIs were reported as risk estimates. To account for non-independence between data rows due to time-splitting of the follow-up periods for each individual, we used a cluster-robust (sandwich) estimator, in which clusters were defined using individual identification numbers.

To control for multiple comparisons, we applied false discovery rate correction (FDR) using the Benjamini–Hochberg procedure [32] and denoted statistical significance (*q* < 0.05) in bold in the tables in the “Results” section.

#### Sensitivity analyses

Although ANGI and BEGIN recruited cases primarily based on AN and OED status respectively, diagnostic crossover is common. To further explore whether the patterns we observed for ANGI vs BEGIN are also observed for AN vs OED diagnostic groups, we performed all above analyses in subgroups of individuals with exclusively AN or OED diagnoses (i.e., no diagnostic crossover) (Supplementary Table S2).

Due to the existence of pleiotropy across psychiatric disorders, the findings may indicate a genetic predisposition to broader psychopathological manifestations instead of being exclusively attributed to schizophrenia. Consequently, we additionally derived a cross-disorder PRS using the same procedure as schizophrenia PRS mentioned earlier, with summary statistics from PGC cross-disorder GWAS [33] and examined the associations between each outcome and the cross-disorder PRS in BEGIN cases. This was not possible for ANGI cases due to overlapping subjects.

## Results

### Baseline description

Table 1 reports the demographic and clinical variables of the study samples. Of 3573 ANGI cases and 696 BEGIN cases, approximately 97% were female with an average age at first ED symptom of 17.3 years old for ANGI cases and 15.4 years old for BEGIN cases. In ANGI cases, the average lowest illness-related BMI was 16.5 kg/m^2^, whereas in BEGIN cases, the average lowest BMI was 19.9 kg/m^2^. ANGI cases had a median CIA score of 16 whereas the median score for the EDE-Q global score was 2.8 in BEGIN cases, with highest scores (median) on shape concern (4.0) and weight concern (3.2) subscales. Within the total follow-up of 86,861 person-years for ANGI cases and 15,340 person-years for BEGIN cases, the most prevalent psychiatric comorbidities were MDD and anxiety disorders for both ANGI and BEGIN cases. No one had received diagnoses of autism or schizophrenia in the BEGIN case sample. More details regarding other characteristics and outcomes including birth year and total diagnoses are described in Table 1.

**Table 1:** Descriptive characteristics of the study population.

	ANGI cases (*N* = 3573)	BEGIN cases (*N* = 696)
Sex female, *N* (%)	3496 (97.8)	678 (97.4)
Birth year, mean (SD)	1988 (6.4)	1990 (7.2)
Age at first ED symptom^a^, mean (SD)	17.3 (4.7)	15.4 (4.0)
Lowest BMI^b^, mean (SD)	16.5 (2.5)	19.9 (3.8)
CIA score, median (IQR)^c^	16 (21)	–
EDE-Q scores, median (IQR)^d^		
Restraint	–	2.0 (3.0)
Eating concern	–	2.0 (2.8)
Weight concern	–	3.2 (3.2)
Shape concern	–	4.0 (3.3)
Global score	–	2.8 (2.7)
Total ED-related diagnoses (mean)	43,664 (12.2)	3858 (5.5)
Total inpatient days related to ED (mean)	129,825 (36.3)	4416 (6.3)
Psychiatric comorbidities, *N* (%)		
Anxiety disorders	1294 (36.2)	237 (34.1)
MDD	1580 (44.2)	262 (37.6)
OCD	300 (8.4)	33 (4.7)
ASD	156 (4.4)	–
ADHD	264 (7.4)	61 (8.8)
SUD	439 (12.3)	74 (10.6)
Schizophrenia	21 (0.6)	–
Total follow-up years	86,861	15,340
Total diagnoses	174,133	23,363
Total unique diagnoses	57,529	9074
Total inpatient days	227,902	14,428

*AN* anorexia nervosa, *ED* eating disorders, *SD* standard deviation, *BMI* body mass index, *IQR* interquartile range, *CIA* clinical impairment assessment, *EDE-Q* eating disorders examination questionnaire, *MDD* major depressive disorder, *OCD* obsessive-compulsive disorder, *ASD* autism spectrum disorder, *ADHD* attention-deficit/hyperactivity disorder, *SUD* substance abuse disorders.

^a^There were 2955 ANGI cases and 646 BEGIN cases with information on age at first eating disorder symptom.

^b^There were 2509 ANGI cases with lowest BMI information, and 640 BEGIN cases with lowest BMI information.

^c^There were 2856 ANGI cases with CIA score information, but this information is not available for BEGIN cases.

^d^There were 639 BEGIN cases with EDE-Q scores’ information, but this information is not available for ANGI cases.

In the sensitivity analyses, which included only AN and OED participants without diagnostic crossover (i.e., no more than one type of EDs was recorded in the registers) (Supplementary Table S2), AN cases had a similar descriptive profile as ANGI cases in terms of age at first ED symptom, lowest BMI, and most prevalent psychiatric comorbidity (i.e., MDD), but with a slightly lower median CIA score of 12 and lower prevalence of psychiatric comorbidities overall (0.1–24.6% compared to 0.6–44.2%). OED cases had a similar descriptive profile as BEGIN cases with the following exception: the average lowest BMI (21.4 kg/m^2^) was higher than in the BEGIN cases.

### Clinical features of EDs

Schizophrenia PRS was significantly associated with clinical severity of EDs measured by CIA and EDE-Q scores for ANGI cases and BEGIN cases, respectively, but in opposite directions. Specifically, one standard deviation increase in schizophrenia PRS was significantly associated with 0.56 decreased CIA scores in ANGI cases and 0.16 increased EDE-Q global scores in BEGIN cases at the conventional significance level of *p* < 0.05. However, the association for CIA scores did not retain statistical significance after FDR correction. EDE-Q subscale scores including shape concern, weight concern, and eating concern were all statistically significantly positively associated with schizophrenia PRS in BEGIN cases after multiple testing corrections, but no statistically significant difference was observed for the restraint subscale scores. Moreover, higher schizophrenia PRS was significantly associated with earlier age at first ED symptom and having more total ED-related hospital contacts (including primary and secondary diagnoses) during the follow-up time in BEGIN cases. The age at first ED symptom increased slightly as schizophrenia PRS increased in ANGI cases, albeit not statistically significantly. Total number of ED-related hospital contacts did not differ for ANGI cases in relation to schizophrenia PRS levels. No significant associations were observed in BMI and total inpatient days due to an ED in either ANGI or BEGIN cases. Additional details regarding the estimated associations between clinical features of EDs and schizophrenia PRS are described in Table 2 including regression coefficients with 95% CIs and corresponding *p* values.

**Table 2:** ED clinical features and schizophrenia polygenic risk score.

Study population	ANGI cases		BEGIN cases	
	Regression coefficient (95% CI)	*p* value	Regression coefficient (95% CI)	*p* value
Age at first ED symptom^a^	0.03 (−0.12, 0.18)	0.713	**−0.35 (−0.64, −0.06)**	**0.018**
Lowest BMI^b^	0.02 (−0.08, 0.12)	0.673	−0.13 (−0.47, 0.21)	0.450
CIA score^c^	−0.56 (−1.04, −0.08)	0.022	–	–
EDE-Q global score^d^	–	–	**0.16 (0.04, 0.29)**	**0.009**
EDE-Q restraint	–	–	0.13 (0.00, 0.27)	0.059
EDE-Q eating concern	–	–	**0.16 (0.03, 0.28)**	**0.015**
EDE-Q shape concern	–	–	**0.18 (0.03, 0.32)**	**0.019**
EDE-Q weight concern	–	–	**0.19 (0.05, 0.34)**	**0.007**
Total ED-related hospital contacts	−0.03 (−0.10,0.04)	0.401	**0.22 (0.05, 0.40)**	**0.012**
Total inpatient days related to ED	−0.04 (−0.17, 0.08)	0.514	0.11 (−0.20, 0.43)	0.481

Bold values denote statistical significance at FDR-adjusted *q* < 0.05.

^a^There were 2955 ANGI cases and 646 BEGIN cases with available information on age at first eating disorder symptom.

^b^There were 2509 ANGI cases and 640 BEGIN cases with lowest BMI information.

^c^There were 2856 individuals with AN with CIA information.

^d^There were 639 individuals with OED with EDE-Q information.

The sensitivity analyses comparing those with AN only or those with OED only revealed a similar pattern for age at first AN diagnosis / first ED symptom, CIA scores, EDE-Q scores, and lowest BMI, despite lacking statistical power (Supplementary Table S3). The sensitivity analyses of clinical features of EDs in BEGIN cases revealed a consistent direction of association with the cross-disorder PRS, similar to that observed with schizophrenia PRS. However, none of the associations with cross-disorder PRS reached statistical significance (Supplementary Table S6).

### Psychiatric comorbidities

Table 3 presents the results obtained from the Cox models testing the risks for psychiatric comorbidities associated with one standard deviation increase in schizophrenia PRS. Higher schizophrenia PRS was associated with a slight but statistically significantly increased risk of comorbid MDD in both ANGI cases [HR (95% CI): 1.07 (1.02–1.13)] and BEGIN cases [1.18 (1.04–1.34)]. Elevated comorbid SUD was also associated with increased level of schizophrenia PRS in both ANGI cases [HR (95% CI): 1.14 (1.03–1.25)] and BEGIN cases [1.36 (1.07–1.73)]. There was a tendency for the risk of all other tested psychiatric comorbidities (except for OCD) to be higher with increasing schizophrenia PRS level. However, the effect sizes were small and statistically non-significant. Results from sensitivity analyses of individuals with AN or OED show the same trend/direction (except for ASD), but statistical significance was not observed (Supplementary Table S4). Intriguingly, the cross-disorder PRS demonstrated a significant association with comorbid MDD and ADHD in BEGIN cases (Supplementary Table S7), while no association was observed with SUD.

**Table 3:** The hazard ratios (HRs) of psychiatric comorbidities among individuals with EDs with different schizophrenia polygenic risk score level.

	ANGI cases		BEGIN cases	
	HR	95% CI	HR	95% CI
Anxiety disorders	1.04	[0.99, 1.10]	1.05	[0.92, 1.20]
MDD	**1.07**	**[1.02, 1.13]**	**1.18**	**[1.04, 1.34]**
OCD	1.00	[0.89, 1.12]	1.03	[0.71, 1.49]
ASD	1.17	[1.00, 1.38]	–	–
ADHD	1.08	[0.95, 1.22]	1.25	[0.96, 1.64]
SUD	**1.14**	**[1.03, 1.25]**	**1.36**	**[1.07, 1.73]**
Schizophrenia	1.34	[0.86, 2.08]	–	–

Bold values denote statistical significance at FDR-adjusted *q* < 0.05.

*ED* eating disorder, *CI* confidence interval, *OCD* obsessive-compulsive disorder, *MDD* major depressive disorder, *ASD* autism spectrum disorder, *ADHD* attention-deficit/hyperactivity disorder, *SUD* substance abuse disorders.

### Cumulative somatic and mental health burden

Table 4 illustrates IRRs for cumulative measurements including total diagnoses, total unique diagnoses, and total inpatient days associated with one standard deviation increase in schizophrenia PRS.

**Table 4:** The incidence rate ratios (IRRs) of cumulative somatic and mental health burden among individuals with EDs with different schizophrenia polygenic risk score level.

	ANGI cases		BEGIN cases	
	IRR	95% CI	IRR	95% CI
Total diagnoses	0.98	[0.93, 1.04]	1.14	[1.01, 1.29]
Total unique diagnoses	0.99	[0.95, 1.03]	1.04	[0.95, 1.13]
Total inpatient days	0.97	[0.88, 1.08]	1.14	[0.96, 1.36]

*IRR* incidence rate ratio, *CI* confidence interval.

Among BEGIN cases, one standard deviation increase in schizophrenia PRS was associated with receiving 14% more diagnoses on average (IRR = 1.14, 95% CI 1.01–1.29). However, this did not withstand the adjusted significance threshold after applying FDR correction. The association between schizophrenia PRS and total inpatient days was as high (IRR = 1.14, 95% CI 0.96–1.36), albeit not statistically significant. No statistically significant association was observed in cumulative health burden with schizophrenia PRS in ANGI cases. In the sensitivity analyses of those with AN or OED only, the patterns were similar to those detected within the full ANGI and BEGIN case samples (Supplementary Table S5). No significant associations were observed between the cross-disorder PRS and cumulative somatic and mental health burden in BEGIN cases (Supplementary Table S8).

## Discussion

Previous studies using molecular genetic approaches and family designs have revealed underlying shared genetic risk between EDs and schizophrenia despite the uncommon co-occurrence of these two disorders in clinical settings [15, 17, 34]. However, few studies have examined clinical outcomes in EDs in relation to schizophrenia or its genetic risk variants.

In the current study, using data from both surveys and national registers, we found schizophrenia PRS to be statistically significantly associated with a higher risk of comorbid MDD and SUD in ANGI cases. Additionally, the schizophrenia PRS showed a negative association with CIA scores in ANGI cases, indicating less severe ED pathology in individuals with higher schizophrenia genetic risk. Similar patterns were observed for AN only cases. These results are in line with previous findings on the impact of schizophrenia family history on clinical outcomes in individuals with AN [18], indicating that common genetic variation associated with schizophrenia contributes to greater psychiatric comorbidity, but less severe ED pathology in individuals with AN. Moreover, BEGIN cases with higher schizophrenia PRS had elevated risk of MDD and SUD, and higher EDE-Q scores, indicating a different pattern of influence for schizophrenia PRS for BEGIN cases compared with ANGI cases, with reference to ED severity. Moreover, results from sensitivity analyses on AN only and OED only cases revealed a similar trend as detected in ANGI vs BEGIN cases, suggesting the different patterns of influence for schizophrenia PRS remain consistent across different diagnostic groups (AN vs OED) in addition to the study differences (ANGI vs BEGIN). These findings strengthen the evidence for shared genetic variants existing between EDs and schizophrenia [15–17, 34], and support the earlier findings of genetic liability to schizophrenia influencing clinical presentations of EDs [18].

### Schizophrenia PRS and psychiatric comorbidities in individuals with ED

It is not unusual to receive other psychiatric disorder diagnoses together with an ED diagnosis [35–37]. Recent studies also highlight the moderate to high genetic overlap across psychiatric disorders [17, 33, 34], and this study supports these close relationships by revealing that ED cases with higher schizophrenia PRS are more likely to have comorbid MDD and SUD. Schizophrenia PRS also has positive associations with all examined psychiatric comorbidities in pure AN and OED groups (except for ASD in the AN only group and OCD in the OED only group) albeit with wide confidence intervals (Supplementary Table S4). Overall, the observed effects were lower than those detected by previous investigations on family history of schizophrenia, which may be due to schizophrenia PRS only capturing part of the genetic risk conferred by common variants while family history of schizophrenia more comprehensively encapsulates the shared genetic and environmental factors in families.

Given the growing evidence of pleiotropy across psychiatric disorders [17, 33, 34], we extended our investigation by testing the cross-disorder PRS in BEGIN cases (Supplementary Tables S6–S8) to explore whether the observed associations reflect a broader genetic predisposition to psychopathology rather than being specifically related to schizophrenia genetic risk components. Remarkably, our findings revealed that the cross-disorder PRS showed significant associations with higher risk of MDD, which is consistent with the associations observed for schizophrenia PRS. Additionally, the cross-disorder PRS displayed an intriguing significant association with ADHD. However, no association was observed between the cross-disorder PRS and SUD (Supplementary Table S7). These findings underscore the complex genetic landscape underlying psychiatric disorders and shed light on the potential overlap and distinctiveness of genetic risk factors influencing specific psychiatric comorbidities in eating disorders.

### Schizophrenia PRS and ED severity

We also found that schizophrenia PRS influenced the severity of EDs in opposite directions in ANGI cases vs BEGIN cases, which is also replicated as a trend in the AN only cases vs OED only cases group (Supplementary Table S3). This partially agrees with our earlier observations, which showed that family history of schizophrenia was associated with less severe EDE-Q scores for individuals with AN [18]. However, we observed different directions of effect for schizophrenia PRS vs family history on EDE-Q scores in OED cases, even though the latter was not statistically significant. Therefore, these results need to be interpreted with caution and further investigation in larger samples is recommended. Furthermore, higher schizophrenia PRS was significantly associated with earlier age at first ED symptom in BEGIN cases (Table 2), which is in line with the previous findings regarding first-degree family history of schizophrenia [18]. No statistically significant result was observed for the lowest BMI, which might be due to an insufficient sample size to detect weak effects. Notably, the cross-disorder PRS was not associated with any clinical features related to ED severity in BEGIN cases (Supplementary Table S6), suggesting that the observed effects may be specially influenced by schizophrenia-specific genetic predisposition.

### Schizophrenia PRS and general health condition

It is interesting to note that every standard deviation increase in schizophrenia PRS was found to be associated with 14% more total diagnoses in BEGIN cases (at the conventional significance level of *p* < 0.05). The effect still held (12%) when restricted to OED only cases (Supplementary Table S5). These results corroborate findings from recent work demonstrating family history of schizophrenia was associated with receiving more total diagnoses in OED cases [18], which further supports the idea that genetic liability for schizophrenia increases the cumulative somatic and mental health burden in individuals with OED. However, contrary to expectations, we did not observe an association between cumulative mental and somatic health burdens and schizophrenia PRS in AN cases. These results differ from the previous study which suggested that AN cases with a family history of schizophrenia had a higher burden of cumulative mental and somatic illness [18]. There are several possible explanations for this result. First, the genetic risk carried by family history of schizophrenia incorporates multiple types of genetic factors. CNVs, rare mutations, common SNPs and their interactions might all contribute to the association detected previously with family history of schizophrenia, while PRS only indexes common SNP risk. Another possible explanation for this is that family history studies cannot distinguish between shared genetic and environmental factors, and the prior observed association could result from a significant shared environmental component.

### Strengths and limitations

The main strength of this study is its unique design; it is the first study to have linked clinical presentations of EDs to schizophrenia PRS to investigate the outcomes of genetic liability of schizophrenia as they developed over time. Still, several limitations must be considered. First, the different patterns observed for ANGI vs BEGIN might be due to study differences including recruitment procedures, different time periods, different illness phases, etc. Our sensitivity analyses suggest that the patterns still hold for pure diagnostic groups (AN vs OED) in addition to study groups (ANGI vs BEGIN) which addressed the issue to some extent. However, study differences cannot be eliminated. Moreover, participation bias still existed in this study for ED severity results (i.e., CIA and EDE-Q scores) which were measured not necessarily during illness phases. Second, the age at first ED symptom was defined from a combination of NPR records and self-report where recall bias might exist. Third, there is considerable heterogeneity in diagnoses within BEGIN which might hinder interpretation. However, sensitivity analyses within the OED only subgroup suggested similar patterns of associations. Moreover, the different coverage period of inpatient and outpatient records within NPR might result in an unbalanced estimate of the cumulative somatic and mental health burden for individuals born earlier or later. Specifically, individuals born in the earlier years of the study cohort might have more unrecorded somatic and mental illness compared to those born in the later years. However, the statistical methods which adjusted for birth year categories in time-split Poisson regression models have addressed this issue to the extent possible. Furthermore, the sample size is moderate. Especially after using a stricter definition for AN and OED, the sample size reduced substantially. Although multiple statistically significant associations were observed in this sample, replication in a larger sample is still needed. Finally, given the well-established sex disparities in the prevalence and clinical presentations of schizophrenia and eating disorders [38–41], as well as recent findings on sex differences in the genetic architecture for both disorders [42–45], it is crucial to explore how sex-specific genetic effects may influence the observed associations in our study. As the majority of participants in our cohorts are women, the associations between schizophrenia PRS and eating disorder outcomes may be influenced by sex-specific genetic effects. Certain genetic variants captured by the schizophrenia PRS may have stronger or weaker effects on eating disorder outcomes in females compared to males, contributing to the observed differences in clinical presentations and treatment responses. Consequently, future research should incorporate investigations of sex-specific effects in the context of genetic risk for both schizophrenia and eating disorders, to gain a comprehensive understanding of the genetic mechanisms underlying the clinical heterogeneity observed in eating disorders.

## Conclusion

Genetic risk for schizophrenia predisposes individuals with EDs to elevated risk of psychiatric comorbidities including MDD and SUD. Higher PRS is associated with the severity of EDs and age at first ED symptom in opposite directions in ANGI vs BEGIN cases (AN vs OED). These findings support the hypothesis that the genetic liability for schizophrenia influences clinical presentations of EDs, and that the patterns in terms of ED-related symptoms differ for AN cases vs OED cases. Further studies would benefit from focusing on how the different types of genetic variation, environmental contributions, and their interactions impact the clinical presentations, comorbidities, and treatment response in individuals with EDs to better understand the mechanisms underlying their clinical heterogeneity.

### Supplementary information

Supplement

## Data Availability

SCZ GWAS summary statistics for PRS computation are available on PGC website (https://pgc.unc.edu/for-researchers/download-results/). Individual-level genetic data from ANGI-(Australia/New Zealand/Sweden/United States) are deposited in dbGaP (http://www-ncbi-nlm-nih-gov.proxy.kib.ki.se/gap) (accession number phs001541.v1.p1). Detailed clinical data from the Swedish population registers are not publicly available due to privacy and ethical restrictions.
