# Supplementary material for: The impact of genetic risk for schizophrenia on eating disorder clinical presentations
Source: Transl Psychiatry. 2023 Nov 29;13:366. doi: 10.1038/s41398-023-02672-3 (PMC10687236; doi:10.1038/s41398-023-02672-3)
Supplement: Supplementary file 1 — Supplement [file 41398_2023_2672_MOESM1_ESM.pdf]

Supplemental Material for

The impact of genetic risk for schizophrenia on eating disorder clinical presentations

Ruyue Zhang[a], PhD, Ralf Kuja-Halkola[a], PhD, Stina Borg[a], Virpi Leppä[a], PhD, Laura M. Thornton[b], PhD, Andreas Birgegård[a], PhD, Cynthia M. Bulik[a,b,c], PhD, Sarah E. Bergen[a], PhD

[a]Department of Medical Epidemiology and Biostatistics, Karolinska Institutet, Stockholm, Sweden
[b]Department of Psychiatry, University of North Carolina at Chapel Hill, Chapel Hill, USA
[c]Department of Nutrition, University of North Carolina at Chapel Hill, Chapel Hill, USA

Table S1. Diagnostic codes used to define psychiatric conditions in the national patient register and the cause of death register

**Diagnoses**	**ICD-9**	**ICD-10**
**AN**	307B	F50.0, F50.1
**OED**	307F	F50.2, F50.3, F50.9
**Schizophrenia**	295A-295H (except 295F), 295W, 295X, V11A	F200–F209 (except F207), F231, F232, F250–F252, F258, F259
**MDD**	296B, 300E, 311	F32.0, F32.1, F32.2, F32.3, F32.8, F32.9, F33.0, F33.1, F33.2, F33.3, F33.4, F33.8, F33.9, F34.8, F34.9, F38.0, F38.1, F38.8, F39
**OCD**	300D	F42.0, F42.1, F42.2, F42.8, F42.9
**Any anxiety disorder**	300A, 300C	F40.0, F40.1, F40.2, F40.8, F40.9, F41.0, F41.1, F41.2, F41.3, F41.8, F41.9
**ASD**	299A	F84.0, F84.1, F84.5
**ADHD**	314	F90.0, F90.1, F90.8, F90.9
**SUD**	303A, 303X, 304A-304H, 304W, 304X, 305A, 305B, 305X,	F10-F19

Abbreviations: AN=anorexia nervosa; OED=other eating disorders; OCD=obsessive-compulsive disorder; MDD=major depressive disorder; ASD=autism spectrum disorder; ADHD=attention deficit hyperactivity disorder; SUD= substance abuse disorders.

Table S2. Descriptive characteristics of the restricted population

	AN case (*N*=1,606)	OED cases (*N*=392)
Sex female, *N* (%)	1,555 (96.8)	384 (98.0)
Birth year, mean (*SD*)	1,987 (6.3)	1,989 (7.2)
Age at first ED symptom [a], mean (*SD*)	17.3 (4.5)	15.6 (4.0)
Lowest BMI [b], mean (*SD*)	16.6 (2.5)	21.4 (3.7)
CIA score, median (IQR) [c]	12 (18.0)	-
EDE-Q scores, median (IQR) [d]		
Restraint	-	2.0 (2.9)
Eating concern	-	2.0 (2.6)
Weight concern	-	3.2 (3.0)
Shape concern	-	4.0 (3.1)
Global score	-	2.8 (2.5)
Total ED related diagnoses (mean)	7,351 (4.6)	1,424 (3.6)
Total inpatient days related to ED (mean)	29,299 (18.2)	380 (1.0)
Psychiatric comorbidities, *N* (%)		
Anxiety disorders	321 (20.0)	120 (30.6)
MDD	395 (24.6)	143 (36.5)
OCD	66 (4.1)	15 (3.8)
ASD	26 (1.6)	-
ADHD	50 (3.1)	33 (8.4)
SUD	95 (5.9)	38 (9.7)
Schizophrenia	2 (0.1)	-
Total follow-up years	40 191	8 925
Total diagnoses	43 114	12 020
Total unique diagnoses	19 151	4 817
Total inpatient days	44 569	5 628

[a] There were 1,110 AN cases with information on age at first AN diagnosis.

[b] There were 945 AN cases and 390 OED cases with lowest BMI information.

[c] There were 1,074 AN cases with CIA score information.

[d] There were 387 OED cases with EDE-Q scores' information.

Abbreviations: AN=anorexia nervosa; OED= other eating disorders; *SD*= standard deviation; BMI= body mass index; IQR= interquartile range; CIA score= clinical impairment assessment score; EDE-Q scores= eating disorders examination questionnaire scores; MDD= major depressive disorder; OCD= obsessive-compulsive disorder; ASD= autism spectrum disorder; ADHD= attention deficit hyperactivity disorder; SUD= substance abuse disorders.

Table S3. Eating disorder (ED) clinical features and schizophrenia polygenic risk score among restricted AN/OED cases.

Study population	AN cases		OED cases	
	Regression coefficient (95% CI)	*p* value	Regression coefficient (95%CI)	*p* value
Age at first ED symptom[a]	0.11 (-0.14, 0.35)	0.394	-0.27 (-0.64, 0.11)	0.166
Lowest BMI [b]	0.007 (-0.15, 0.17)	0.937	-0.06 (-0.48, 0.36)	0.777
CIA score[c]	-0.74 (-1.46, -0.01)	0.047	-	-
EDE-Q global score[d]	-	-	0.10 (-0.06, 0.25)	0.211
EDE-Q restraint[d]	-	-	0.06 (-0.12, 0.23)	0.529
EDE-Q eating concern[d]	-	-	0.10 (-0.05, 0.26)	0.197
EDE-Q shape concern[d]	-	-	0.10 (-0.08, 0.28)	0.265
EDE-Q weight concern[d]	-	-	0.13 (-0.04, 0.30)	0.145
Total eating disorder related hospital contacts	-0.05 (-0.19,0.09)	0.484	0.19 (-0.03, 0.41)	0.089
Total inpatient days related to eating disorder	0.02 (-0.25, 0.29)	0.880	-0.19 (-0.86, 0.49)	0.592

[a] There were 1231 AN cases with available information on age at first diagnosis.

[b] There were 1082 AN cases and 390 OED cases with lowest BMI information.

[c] There were 1224 AN cases with CIA information.

[d] There were 387 OED cases with EDE-Q information.

Table S4. The hazard ratios (HRs) of psychiatric comorbidities among restricted AN/OED cases with different schizophrenia polygenic risk score level.

	AN cases		OED cases	
	HR	95% CI	HR	95% CI
Anxiety disorders	1.09	[0.98, 1.23]	1.04	[0.86, 1.26]
MDD	1.11	[1.00, 1.23]	1.09	[0.92, 1.29]
OCD	1.01	[0.78, 1.31]	0.94	[0.55, 1.62]
ASD	0.95	[0.63, 1.42]	-	-
ADHD	1.17	[0.88, 1.56]	1.32	[0.92, 1.91]
SUD	1.16	[0.94, 1.43]	1.14	[0.82, 1.58]

Abbreviations: AN= anorexia nervosa; OED= other eating disorder; HR= hazard ratio; CI= confidence interval; OCD=obsessive-compulsive disorder; MDD=major depressive disorder; ASD=autism spectrum disorder; ADHD=attention deficit hyperactivity disorder; SUD= substance abuse disorders.

Table S5. The incidence rate ratios (IRRs) of cumulative somatic and mental health burden among restricted AN/OED cases with different schizophrenia polygenic risk score level.

	AN cases		OED cases	
	IRR	95% CI	IRR	95% CI
Total diagnoses	0.99	[0.92, 1.07]	1.12	[0.94, 1.34]
Total unique diagnoses	0.98	[0.93, 1.04]	1.02	[0.90, 1.16]
Total inpatient days	1.01	[0.84, 1.22]	1.13	[0.88, 1.44]

Abbreviations: AN= anorexia nervosa; OED= other eating disorders; IRR= incidence rate ratio; CI= confidence interval.

Table S6. ED clinical features and cross-disorder polygenic risk score in BEGIN cases.

	Regression coefficient (95%CI)	*p* value
Age at first ED symptom [1]	-0.24 (-0.52, 0.04)	0.096
Lowest BMI [2]	-0.002 (-0.32, 0.32)	0.988
EDE-Q global score [3]	0.12 (-0.01, 0.24)	0.061
EDE-Q restraint	0.10 (-0.03, 0.23)	0.116
EDE-Q eating concern	0.10 (-0.03, 0.22)	0.097
EDE-Q shape concern	0.12 (-0.03, 0.27)	0.107
EDE-Q weight concern	0.15 (0.01, 0.29)	0.041
Total ED related hospital contacts	0.15 (-0.02, 0.32)	0.092
Total inpatient days related to ED	0.34 (-0.10, 0.78)	0.130

[1] There were 646 BEGIN cases with available information on age at first eating disorder symptom.

[2] There were 640 BEGIN cases with lowest BMI information.

[3] There were 639 individuals with OED with EDE-Q information.

Table S7. The hazard ratios (HRs) of psychiatric comorbidities among individuals with EDs with different cross-disorder polygenic risk score level in BEGIN cases.

	HR	95% CI
Anxiety disorders	1.07	[0.94, 1.22]
MDD	**1.17**	**[1.04, 1.33]**
OCD	1.10	[0.77, 1.56]
ADHD	**1.40**	**[1.07, 1.81]**
SUD	1.00	[0.79, 1.27]

Abbreviations: ED=eating disorder; CI= confidence interval; OCD=obsessive-compulsive disorder; MDD=major depressive disorder; ADHD=attention deficit hyperactivity disorder; SUD= substance abuse disorders.

Bold values denote statistical significance at FDR adjusted q< 0.05.

Table S8. The incidence rate ratios (IRRs) of cumulative somatic and mental health burden among individuals with EDs with different cross-disorder polygenic risk score level in BEGIN cases.

	IRR	95% CI
Total diagnoses	1.11	[0.98, 1.26]
Total unique diagnoses	1.04	[0.95, 1.13]
Total inpatient days	1.17	[0.91, 1.49]

Abbreviations: IRR= incidence rate ratio; CI= confidence interval.
